# A Description of the Different Grades of Bilious Remittent Fever, as They Occur in the Wabash Valley, and Particularly in Clark County, Illinois

**Published:** 1842-01

**Authors:** James Smick

**Affiliations:** Darwin, Illinois


Art. VI.—*A Description of the different grades of Bilious Remittent Fever, as they occur in the Wabash Valley and particularly in Clark county, Illinois.* By James Smick, M. D., of Darwin.

The valley of the Wabash furnishes the most extensive field for observation on the grades of bilious fever, with which I am acquainted; and it may not perhaps be uninteresting to the medical profession for me to state what my experience has taught me on that subject during a residence of eleven years in Darwin, Clark county, Illinois.

Topography.—The Wabash is a beautiful river, running generally south. Its banks are very low, and extend back from one to three miles. Darwin is situated on a bluff bank on the west side of the river about eighteen feet above high water mark, at the upper end of Walnut prairie which stretches southward from it. At the south end of the prairie, Mill Creek empties into the river about six miles below Darwin. The ground for three or four miles above its mouth is very low, and liable to be overflowed at a high stage of water. East of Darwin on the opposite side of the river is a low bottom four miles wide. The land next to the river is higher than it is back. Several small bayous lead back to the lower grounds. North of Darwin about 5 miles, Big Creek empties into the river. The land about its mouth is for some distance very low also, and is overflowed as well as about the mouths of some smaller streams which empty between it and Darwin. West of Darwin about a mile we meet a low strip of land which bounds the prairie westward and encircles it. There are also on the prairie some bayous leading from the river to basins, forming ponds. In a high stage of water these are large, covering many acres and are never entirely dry. The face of the country is generally level. The soil is alluvial and very rich, consisting of sand and black loam. There is a much greater proportion of sand in the soil of the high prairie than of the low lands which are overflowed. The bottoms are covered with a heavy growth of timber, such as is peculiar to a very rich soil. On the edges of the prairie it is shrubby in consequence of the annual burning of the prairie before it was settled. Such of the above described lands as are overflowed are, after the month of May, covered with a very rank growth of vegetation of every kind. Such of the prairie as is not under cultivation is covered with a heavy growth of prairie grass, sometimes six or eight feet high. The low strip of land, west of the prairie, between the breaks of Big and Mill creeks, is what we call barrens. The soil is of a more argillaceous nature and consequently very wet. Though I have here described a limited tract of country, yet the general features of this description apply with but little variation to the whole valley of the Wabash through its length, so far as I have travelled over it. The general character, too, of the diseases is the same through the whole valley.

We generally have a very high stage of water sometime during the months of March, April, or the first part of May. This general rule has never failed since my residence here but in one year, 1833, when we had no high water until the last of June. Some years we have two freshets. In 1834 and 1836, the second came in the last of May and first of June. In 1837 the second rise came in July.

We have more or less of every grade of fever from the simplest intermittent to the most fatal remittent, every year. But in those years in which there has been a late rise of the river we had an unusual number of cases of the severest grade. Our sickly season, as it is familiarly called here, begins about the 1st of July and continues until the frosts in the fall. Usually in the latter part of June and the first of July cases of diarrhœa and cholera-morbus occur; then some of the milder cases of remittent fever; and about the first of August the severe grade make their appearance.

Predisposing Causes.—Among the predisposing causes the first and principal is marsh-miasma. All that is necessary to produce it exists abundantly every season, i. e., heat, moisture, and vegetable decomposition, but most abundantly in those seasons in which the river overflows its banks after vegetation has sprung up in the bottoms. Then, after the water has retired, the smell of decaying vegetable matter is very perceptible. The heavy growth of timber upon the bottom lands delays the process of decomposition, as it presents the full action of the sun upon the decaying vegetation and prevents the escape of miasmata already generated. In those seasons when we have a late rise, we have not only an additional number of cases, but an increased malignancy in the character of the disease. This fact, together with the smell which in those seasons is so distinguishable, indicates the real source of malaria. In those seasons congestive and hepatic modification of bilious fever were by far the most common, and the cases were the most numerous, every family suffering more or less—the old settlers being the most exempt.

In those seasons in which the river takes no late rise, sickness is confined to the neighborhood of the ponds, to those spots where a large quantity of grass grows in the river, or where the current is not swift enough to carry off all decaying matter, and to the neighborhood of mill ponds. The turning of a heavy coat of prairie grass under in the fall and opening it in the spring to the action of the sun is, I think, a very fruitful cause of miasma. It would seem also that corn, when growing very rank, throws out malaira; for those families whose houses are closely surrounded by it on all sides are sick more or less every year. Where the bottom logs of a house are in a state of decay, or the floor rests upon the ground, the inhabitants suffer every year. Our summers are warm, the thermometer standing often as high as 96°. Probably those rainy summers in which the river takes a second rise, are not as warm as the drier ones, though here I cannot speak positively as I have no thermometer. Exposure upon the prairie to the heat of the sun and the reflection of this heat from the sand, stimulating the liver to morbid action, is a predisposing cause of fever.

Persons coming from a healthy region, or where there is little of the chill and fever and residing on the Wabash amidst these various causes of disease, are universally sick the first or second year after their removal, unless they are laboring under some chronic disease. If this is the case their health usually improves. Nervous temperaments are not so liable to fever here as bilious and sanguine.

Exciting Causes.—Among these may be reckoned overloading the stomach with indigestible food or with such as will sour; exposure to the north or north-east wind which is quite cold even in the month of August. The change occasioned by the rise of this wind is very sudden sometimes, and very great. The thermometer may, in two hours, sink from 90° to 60°. Such a sudden reduction of heat in the surrounding air throws the fluids in from the surface upon the internal organs of the system. I think such exposure the most common cause of relapse. Any sudden check of perspiration operates as an exciting cause; the drinking of old drinks when the system is preternaturally heated—sleeping with open windows and doors; marsh miasma becomes an exciting cause when applied in this way. Fatigue may justly be reckoned another of these causes.

Prophylactics.—A residence should always be chosen on the west or south-west side of marshes, or of the river, and a skirt of timber should be left between the house and the source of miasma, where it exists naturally; and if not, one should be planted so as to intercept the miasma. Where it is possible ponds should be drained. And where the banks of the river will permit levees should be thown across the mouths of the bayous, to prevent the influx of water during a fresh. The under-growth should be cut out of the low timbered bottoms, and a sufficient number of cattle kept in them to destroy the growth of the wild vegetation, until the tame grasses can be made completely to sod them. Houses without cellars should be sufficiently elevated to admit a current of air under them. A heavy growth should not be permitted in the yard of any kind of vegetation, with the exception of blue grass and shade trees. Corn should not grow very near.

I think that if the above hints are appreciated by the people of the Wabash as they deserve to be, and are once acted upon, we may expect to enjoy a freedom from such sickness as now yearly visits us. Those whose bowels are costive or who are subject to a bilious purging, should take a dose of blue pills as often as is necessary to correct either, and carefully avoid all crude indigestible articles of food and any exposure to the cold north or north-east winds.

Pathology.—I do not expect, under this head, to discuss the various theories that exist upon this subject, but to give what I conceive to be the true pathology of bilious fever, from the observations which my limited means have enabled me to make. Both the anatomical theory of Broussias and his followers, and the congestive theory of Armstrong and his followers are founded in error; taking an effect of the fever for the cause. The reasoning by which these great theories are sustained, is too well known to the profession for me to mention it here. My own ideas cannot be better expressed than in the words of Prof. Yandell, occurring in his essay on bilious fever in the Western Journal of Medicine and Surgery, September number, 1841, page 179: "The malaria exerts its first influence upon the nervous system. The nerves of the cerebro-spinal axis are first disturbed. Hence the languor and general malaise, the precursors of an attack of fever. The nervous system is marked by a peculiar law—that of periodicity or intermission. It is active, and reposes by turns. Repose is essential to its health. Intermission being an attribute of the tissue which sustains the first morbid impression, the same character is stamped upon the disease. All essential fevers exhibit this tendency, and in one type, the intermission is complete. All diseases located in the nervous system are intermittent, notwithstanding that the cause may be permanent. A spicula of bone, for example, irritating the brain may excite epilepsy recurring at distant intervals, though the irritation be always present. And, in like manner, coma, catalepsy and hysteria recur periodically, while the cause of irritation may be some permanent organic lesion. In fever, the system of nerves controlling animal life being depressed, all parts of the system—the skin, the viscera, and all the organs of circulation—are disturbed with it. For it is not mere depression of action; it is action also perverted. Congestions of the viscera, abdominal, thoracic, and cerebral, occur, which changes of temperature, irregularities of all sorts, fatigue, etc., especially favor; and these congestions being repeated, to be followed, still, by violent reaction, lesions at length are established in some system of organs, and become independent foci of irritation, reacting upon the cerebro-spinal axis, and increasing and perpetuating the morbid commotion." This constitutes the first class of phenomena in fever and "the repetition and excessive amount of the local congestions recurring in the cold stages of fevers, give rise to a hyperæmic condition of the affected organs. This condition frequently excited leads at least to local inflammation. New local inflammations are set up in various organs, and those which, in the beginning were only the effect of disease, become by reaction on the system the cause of its continuance." In one case it produces inflammation of the mucous surface of the stomach and bowels during the continuance of the febrile excitement. This condition commences at the first with the chill and continues only until the fever subsides; but after a few paroxysms the inflammation lasts all the time. This constitutes what may be termed gastric remittent fever, and in another case it may produce the same effects on the stomach and bowels, deep-seated congestion in various organs, followed by a broken reaction and in some cases by no reaction at all. This constitutes what is usually termed congestive fever. And in other cases, a more violent reaction follows the chill, which assumes a continued form, with violent congestion of the liver, which seems in some cases completely locked up, and in others to take on a morbid action, throwing out a large quantity of irritated bile with great oppression of the ganglionic plexus of nerves supplying the liver. This constitutes what may be termed the hepatic modification of bilious fever.

Thus we have varieties of bilious remittent fever, marked by sufficiently distinct symptoms, which for therapeutical purposes should be considered under different heads.

Perhaps the advocates of the anatomical theory examined cases of the first two varieties above mentioned, which would give, on a post-mortem examination, the appearances which they claim. And perhaps the advocates of the congestive theory examined only cases of the last variety, in support of their theory. And this may be the reason why they have not better argued. I make this as a mere suggestion, hoping it may excite the attention of some one whose opportunities will enable him to make the observations necessary in order to arrive at correct conclusions on this subject. So far as my observation relative to the different varieties of bilious fever, as it has occurred here, in the Wabash valley, during an extensive practice of eleven years, has extended, all the cases which have come within my notice have assumed some one of the above forms, with the exception of the simple intermittent and the common bilious remittent fever of authors. That I may be better understood, I will treat each class separately.

1st. Gastric Modification of Bilious Remittent Fever.—This is the mildest of the three varieties. It commences with a chill followed by a fever, and in its mildest form, a slight fever occurs on the evening of the next day after the attack, lasting until the recurrence of the chill on the third day. After a few paroxysms in this way, usually at the commencement of the third chill, a severe vomiting comes on and continues until reaction is fairly established, which is after about three hours, and then a purging succeeds, which continues as long as the excitement lasts. In the first paroxysm, which is attended with vomiting, the drinks that are taken, and in some cases bile, are all that is thrown up. But in a majority of cases during the succeeding paroxysms, blood is both vomited and purged, in some cases abundantly, in others in moderate quantity. At this stage the patient complains of great heat at the stomach, with an intolerable thirst; and if drinks are taken to satisfy it they are immediately rejected. In a majority of the cases, during the continuance of the purging, severe pains are complained of through the whole region of the bowels. These last mentioned symptoms are experienced only in the paroxysms which are preceded by a chill. When this variety takes on the severest form, the chill is of the quotidian type, and of course the respite between the paroxysms is not so long; and the disease terminates sooner. If it does not prove fatal before the ninth day, as it dose in the severe form, unless arrested by medicine; it assumes the form of diarrhœa attended with chronic gastro-enteritic inflammation, which in most cases yields very slowly to medicine. The pulse is very rapid, but in general not very strong during the whole course of the disease, but more especially so after the vomiting and purging of blood takes place. The heat of the surface during the fever is very considerable, but in most cases terminates in a gentle, and in some cases, a profuse perspiration.

The tongue is oftenest covered with a white coating, sometimes, however, with a yellow one; during the first few paroxysms it is moist, then it becomes dry during the paroxysm, and when the disease assumes the chronic form mentioned above, it is covered with a dry brown coat. The breathing during the paroxysms is very irregular. Just before vomiting it is very quick and somewhat laborious; and immediately after, or while the patient is free from extreme sickness at the stomach, it is quite easy, and sometimes rather slow. The countenance expresses considerable anxiety; the features are sharp; the eyes are sunken; and when the disease continues long, they become inflamed. The patient sleeps with them partly open, and before a fatal termination, they are almost entirely open and turned upwards and back. Subsultus tendinum sometimes attends the last stage, but not so frequently as in some of the other forms. An irresistible disposition to sleep comes on before death, from which the patient frequently starts as if alarmed.

I regard as the most unfavorable symptoms, a copious watery or bloody purging, which is not easily controlled by medicines—the patient's showing by moaning, talking in sleep, and other signs that he is in considerable pain, while if asked he replies that he feels well—a brown dry tongue; small feeble pulse, and somnolency amounting in some cases to complete stupor. In 1833 and 1834 the vomiting and purging were, in a majority of cases, attended with severe cramping of the arms and legs and abdomen; since then it occurs more rarely, but attends in some cases every year. This form of fever sometimes runs into the congestive form, especially if emetics of tartar are used.

*Diagnosis.*—This form of fever is distinguished, first, from the congestive form by its having a regular stage of excitement after each chill and less sweating. The surface is more full than in the congestive form—not so contracted. The tongue is also drier. This form is attended also with more pain, especially in the bowels, and with more acute feeling generally than the congestive variety.

Secondly; it is distinguished from the hepatic variety by its having a chill before every other and in some cases every paroxysm, with an intermission in some cases, and in others, a distinct remission. The skin and conjunctiva of the eyes are not so yellow in this form as in the hepatic.

*Treatment.*—In this variety there are two great indications of cure: First, to allay the irritation of the stomach and bowels, and moderate the excessive febrile excitement; and, secondly, during the remission or intermission, if there be one, to prevent the recurrence of the next paroxysm. To accomplish the first, should there be much vomiting, I give a draught of soda water in a state of effervescence, with 18 or 20 drops of laudanum in it, immediately after each vomiting. If this should not be sufficient to stop it, give 20 drops of laudanum with 6 drops of essence of peppermint every two hours; apply also a sinapism to the pit of the stomach. Where the above remedies do not lie upon the stomach long enough to have the desired effect, apply a cloth wet in laudanum, or opium moistened sufficiently to admit of its being spread, to the pit of the stomach, after the mustard seed poultice is taken off. Let this remain as long as there is any sickness at the stomach. And to moderate the febrile excitement, I give a teaspoonful of spirits nitre dulcis every two hours, and sponge the surface with equal parts of warm vinegar and water, or weak soap-suds warm, as often as the heat is considerable. Applied warm it has a much better effect then when applied cold. It hastens the third or sweating stage, which terminates the paroxysm. It should be applied freely and often—as often as is necessary to keep down the excessive heat. After the irritation is sufficiently subsided, give powders composed of 6 grains of calomel and $\frac{1}{4}$ grain of opium, where there is much purging $\frac{1}{2}$ grain, every three hours until three or four are taken. If they do not operate sufficiently within four or five hours after the last is taken, give a dose of castor oil or rhubarb, sufficient to operate. As soon as the operations become watery and large, check the purging with laudanum or opium in substance. If the chill should recur while the system is under the operation of the physic, the vomiting and purging will be much greater in the next paroxysm; therefore, the operation of it should be checked always before it is expected. I follow this course in each paroxysm, until the secretions in general become free and healthy, especially from the liver, or the fever is checked by means hereafter mentioned, to be used during the remission or intermission. Where the patient has been once salivated, or the constitution is easily affected by calomel, its effects should be closely watched upon the glandular system and its use never continued long enough to produce ptyalism, if it can be avoided. I have never seen any good, but on the contrary, much inconvenience, as the result of such a state. Calomel in small unirritating doses, repeated at short intervals, worked off by some mild cathartic, at a time when the system is freest from irritation, while its effects upon the glandular system are fully ascertained before more is given, produces a much better effect in this and the congestive form of fever, according to my experience, than when given in large doses.

The effect produced by it is more under the control of the physician. The use of opium under any circumstances, during the febrile excitement, is, I know, strongly objected to by almost all writers on this subject. The successful operation of the other remedies depends upon the promptness with which the irritation of the stomach and bowels is allayed; and I have found no medicine to answer this purpose, in this and the congestive variety of fever, better than opium or laudanum. The injury said to be produced by it upon the brain, I have never seen to follow its use. The delirium is no greater after than before it was given, but the ease experienced by the sufferer is almost indescribable. The torpor of the bowels, feared from its use, does not follow when it is given as directed above. It is to be given only where the vomiting and purging are too severe to be permitted to continue; and these are to be controlled at any time regardless of the fever.

To answer the second grand indication, in recent cases and all others at any stage, where the symptoms indicate no inflammation of the stomach and bowels, or any other important organ, during the remission I give large doses of quinine in the paroxysm that is preceded by a chill, just before it comes on: say from 1 to 10 grains, varying the dose according to the age and condition of the patient. My usual portion is 6 grains for an adult. The first dose of this size should be given about four hours before the chill is expected; and a second, one hour before the chill. And in cases where the disease has existed some days, or where the chill is of the quotidian type, a portion should be given every six hours, until the time for the next regular paroxysm has passed. This is necessary in order to prevent the recurrence of the chill, which sometimes supervenes eight hours after its usual time, in cases where this precaution is not taken. In recent cases this is unnecessary. The last portion may be given two hours before the time of the regular paroxysm.

Large doses of quinine have succeeded in my hands far better than smaller ones, both in this and the congestive form of fever. In cases where the tenderness of the bowels on pressure, the dry tongue, and the excitement continuing from chill to chill contraindicate the use of quinine, I give a powder composed of 3 grains of gum camphor, 4 grains of calomel and $\frac{2}{3}$ grain of opium once in three hours, commencing seven hours before the chill is expected, until three of them are given. These should be repeated, commencing seven hours before the recurrence of the next chill. In most cases the first course will check the chill, but the fever is apt to rise at about its usual period, but it also disappears after the second course of powders. When these camphor powders are used during the remission, the calomel and opium powders directed to be used in the stage of excitement are to be omitted. In their stead a cathartic of castor oil may be administered, whose operation should be over when the time arrives for the use of the camphor powders. This cathartic should follow each course of powders, unless the bowels are sufficiently moved without it.

When the disease assumes the chronic form, a pill may be given composed of 1 grain opium, 4 grains calomel, and 2 grains ipecac. once in three hours until four are taken, following them in about four hours with a dose of castor oil and 8 or 10 drops of turpentine, unless the bowels are sufficiently moved without.

These pills followed by the oil and turpentine should be repeated every twenty-four hours, until the operations become healthy, and the pain and tenderness of the epigastric region, and the excessive purging are entirely relived. As soon as the calomel in the above receipt affects the mouth, it should be discontinued, while the other medicines are to be still given. The drinks should be gum water, slippery elm water and the like. If the case does not yield readily to the above means, a blister should be applied to the bowels, sufficiently large to cover the whole seat of the pain. Where there is a copious bloody purging, large doses of opium have in my hands had the best effects in stopping it. In severe cases I have given with the happiest effects, as much as 4 grains every four hours, but the dose should be varied to suit existing circumstances. When large quantities of yellow bile are vomited, laudanum will not arrest it; two or three swallows of water as hot as it can be taken, drank occasionally, and after each spell of vomiting, will succeed in so reducing the quantity of bile that laudanum will arrest it entirely.

2dly. THE CONGESTIVE VARIETY OF BILIOUS REMITTENT FEVER.—This is a more fatal form of fever than the preceding. It is nothing more nor less than the "algid intermittent" of Alibert. Its type is the same as that of the preceding variety, except that the chill oftener precedes fever every day than in the former. The chill continues from one to six hours before the excitement follows; this, though sometimes considerable, does not in the majority of cases rise very high. In many cases the patient complains of great heat of the surface, while the skin continues only cold. In most instances there is a copious perspiration standing in great drops on many parts of the system, warm where the surface is warm and cold where it is cold. Sometimes this perspiration appears only while the sickness is great. There is always great irritation of the stomach and bowels. Vomiting usually commences with the chill and continues at intervals through the paroxysm if not prevented by medicine. The fluid vomited is for the most part the drinks that are taken, sometimes accompanied with blood. Sometimes there is in the vomited fluid a substance resembling small pieces of flesh floating. The operations from the bowels are very large and watery and tinged with bile. In other instances clear blood is vomited, and in others still a fluid resembling the washings of meat, or sometimes of a whitish or rather milky color. The countenance is ghastly and very expressive of anxiety, the lips generally blue, the eyes deeply sunken, and the tongue moist and generally not very thickly furred, and livid spots appear on various parts of the body.

The senses are not much affected, sometimes indeed the intellectual faculties are rather acute and the patient disposed to talk a great deal. The voice is hollow and sometimes reduced to a whisper. Occasionally indeed we see them become delirius as soon as the fever rises, and sink into a state of complete stupor from which it is difficult to arouse them until the paroxysm has passed. In such cases there is less purging than in the others, and less sweating until the fever begins to subside. The breathing, when the surface is covered with a cold sweat and the mind is not much affected, is frequently made by a long deep sigh; and when stupor prevails is more laborious still. The patient is generally very restless, tosses about a great deal, but does not suffer much pain. Incoherent speech, subsultus tendinum, involuntary discharges of the bowels and a marble coldness of the extremities, are symptoms which generally precede a fatal termination.

*Diagnosis.*—In this variety the excitement which follows the chill is not so regular. The surface, especially the extremities, are much cooler and covered with a more profuse perspiration. The tongue is also more moist than it is in the former variety. It is distinguished from the hepatic variety by a more general perspiration, and a less excitement, and a marked state of intermission or remission.

*Treatment.*—In this variety we have three great indications of cure; first, to allay the irritation of the stomach and bowels. Second, to moderate the excitement where that exists, and where the reaction is broken or not established, to produce an equilibrium of excitement through the system in the paroxysm; and, Thirdly, during the intermission or remission to prevent a recurrence of the paroxysm.

To fulfil the first I use about the same remedies recommended for this purpose in the gastric variety, viz: laudanum in soda water, or peppermint and laudanum, the sinapism to the stomach, and the application of moistened opium after the mustard seed poultice is taken off. These must be varied to suit existing circumstances. After the irritation is in some degree allayed, a general reaction sometimes follows without the use of revulsives. It is therefore absolutely necessary that this should be sufficiently allayed to check the vomiting and purging, or our effort to produce reaction will be unavailing. After the vomiting and purging are sufficiently allayed, a powder composed of 1 gr. opium, 6 grs. calomel and 2 grs. camphor should be given every four hours until four are taken. And in those cases where the intermission is not sufficiently long to admit the use of quinine, according to directions to be given, the powders should be so administered that the last one will be taken about an hour before the recurrence of the paroxysm. Cathartics should be used very carefully in this form of fever, but if the bowels should not be moved within six or eight hours after the last powder is taken, let oil be given if it can fairly operate before another chill is expected; if not, defer it until the chill has passed and its accompanying irritation has subsided. I generally combine camphor with all the calomel which I give in this form; it seems to facilitate its action upon the system, causing it to produce the desired effect sooner. In severe cases I repeat the powders every twenty-four hours, and in milder ones every thirty-six hours until the discharges show a healthy state of secretion, especially from the bowels, and from the liver, or until the calomel affects the mouth. A cathartic should follow each course of powders unless the bowels are sufficiently moved without it. To moderate the excitement, where that is excessive, the surface should be frequently sponged with warm vinegar and water as in the former variety. Where the reaction is broken let the surface be rubbed with warm camphor and warm vinegar with cayenne pepper boiled in it alternately. The rubbing should be resorted to every few minutes; it should be brisk and done under the bed-clothes. And should the extremities be very cold they should be wiped off dry and wrapped in warm dry flannels after each rubbing. Warm brandy may also be rubbed on with advantage. This rubbing should be diligently maintained in cases requiring it until reaction is fairly established. But if the system should run into a complete state of collapse, or should it be in that state when the physician is called, the "cold water dash" should be resorted to. A quantity of water should be poured from a pitcher or small pail for a minute or two upon the surface, which should then be wiped off dry as quick as possible and wrapped in warm blankets. A little brandy toddy sweetened well with loaf sugar should be given immediately after, and if the purging is great, 25 or 30 drops of laudanum. It is important too that the rubbing, spoken of above, should be kept up from time to time under the clothes. If reaction does not ensue in an hour and a half after the application of water, let it be repeated in the same manner. This will succeed when no other remedy will, and should never be neglected in the asphyxiated stage. After reaction is fairly established the treatment before recommended should be pursued. The third indication of cure is to prevent if possible the recurrence of the chill, and to secure this result large doses of quinine answer better than any and all other remedies that my ingenuity could invent, or that I have ever seen recommended by authors for this purpose. Indeed it is the only thing that can be relied upon, and even that, in some cases, fails. As soon as the remission or intermission is sufficiently distinct, it should be given in 10 gr. doses every six hours, and so timed that the last powder shall be taken about an hour before the chill is expected. And if there should be an error in the calculation of the time and the chill should return sooner than it was expected, a portion should be given as soon as the symptoms of its return are felt. It should be resorted to in each interval of fever, unless some symptom should contra-indicate its use—such as a stomach too irritable to receive and retain it, severe pain in the stomach, or pain in the head with large bloody discharges from the bowels. In such cases, which are but few, it should be omitted and the camphor powders mentioned above should be given before the chill, so that the system may be under their influence when its time arrives.

When the patient sinks into a state of stupor, I apply a blister to the back of the neck, baths of mustard seed to the soles of the feet, and wet the head frequently with spirits of camphor or vinegar and water, in addition to other remedies. Whenever large quantities of clear blood or bloody water are discharged from the bowels, opium pills in 2 gr. doses should be given every three hours until it is stopped. After the patient becomes convalescent, which is between the third and seventh day, he should take an occasional dose of blue pills to keep the bowels regular, and some tonic mixture to restore the tone of the stomach, either small doses of quinine or a bitter made of Peruvian barks, gentian and rhubarb in wine or brandy, and taken three or four times a day for some days This facilitates the recovery very much, and at the same time tends to prevent a relapse, a thing very apt to occur both after this form of fever and the gastric variety.

3dly. The Hepatic Modification of Bilious Fever.—This form so far as my observation goes is by far the most fatal one: it is very malignant in its character. It may very justly be compared to a "dog that bites without barking." Its attack is very insidious. The patient complains for several days of not feeling very well, and in the evening has a little fever, but not sufficient often even to attract his attention, much less to cause him to take medicine. It may exist a week in this way before it is fully developed. In such an attack it is not preceded by a chill, but often its approach is more sudden, commencing with a short chill followed by a high fever that takes on the continued form. In some instances there are evening exacerbations, and slight morning remissions growing less and less distinct; in others the exacerbations last from 1 o'clock to 6 in the evening, and from 1 o'clock to 6 in the morning with evening and morning remissions, showing two distinct paroxysms in 24 hours. Sometimes these paroxysms are preceded by slight chilly sensations in the back and some little coldness of the fingers and toes. Generally a severe pain is felt in the forehead immediately above the eyes and in the back part of the head, and a severe aching in the knee joints and bones generally. The pulse is at first quick and generally strong, and sometimes hard, but after the disease takes on a typhoid type, it is very frequent, beating as often as 150 a minute; sometimes rather intermittent, at others weak. During the exacerbation strong pulsation can be perceived in the carotid and temporal arteries at some distance from the bed-side. A throbbing can be distinctly felt in the right hypochondrium and epigastrium. The surface is at first of a red and sometimes of a yellow hue, but does not usually assume the yellow appearance before the third or fifth day. The cheeks are at first flushed, but after the seventh day, sometimes later, they assume a cadaverous appearance. The countenance at this time is very ghastly, the face hippocratic. There is a great restlessness, especially after the irritable excitement comes on, in the typhoid stage. The tongue is at first coated over with a thick yellow fur, it is at first moist but gradually becomes dry, and as the moisture leaves it its color changes to brown and sometimes to black with a depression in the centre. While dry it cracks considerably and in some cases bleeds. The patient is sometimes unable to protrude it beyond the teeth. The voice is at this time very mumbling.

The patient complains of considerable sickness from the commencement, and some vomit occasionally; in others the stomach contracts forcibly once or twice, throwing up a considerable quantity of fluid without being much sick. This fluid is mostly the drinks that have been taken, occasionally, however, it is green bile. This latter I consider a very dangerous symptom. The discharges from the bowels are of a muddy brown, sometimes of a green color, and generally very watery. Towards the last the liver throws out a quantity of pitchy, black bile. The urine is highly colored. There is but little sweating in this form at first, and it is confined entirely to the face and breast, but about the seventh day or ninth it becomes more general. The symptoms which denote a fatal termination are subsultus tendinum—the patient's endeavoring to rise from the bed without assigning any reason for it; incoherent speech; double vision or the loss of vision; involuntary discharges of a dark pitchy matter; laborious breathing, especially in making an inspiration; cold, clammy sweat. Death supervenes usually between the seventh and eleventh days, sometimes not until the 21st.

This fever, in its commencement, is of the inflammatory diathesis, but if not broken up before the seventh or occasionally the ninth day, it assumes a typhoid character, which is attended with a diarrhœa, a peculiar irritable excitement, great restlessness, the patient showing signs of considerable distress, but at the same time professing to feel well, much better when interrogated, small, feeble but frequent pulse, a tremulous tongue, great somnolency, &c.

This form can be so easily distinguished from either of the other two by the diagnoses given in treating of them, and by its own enumerated symptoms as to render a particular diagnostic essay unnecessary.

There are three grand indications of cure: First, to allay the arterial and febrile excitement. Second, to open the portal system where it is locked, and to change the irrtiated state of secretion generally; and thirdly, to prevent its running into a typhoid state. When there is a hard pulse, pain in the head, and a torpid state of the bowels, general blood-letting is indispensable, and should be resorted to in the height of the paroxysm, to such an extent as to overcome entirely the hardness of the pulse, and afterwards repeated at any time when the hardness returns. Great care however is necessary, and a close observation of its effects. Much bleeding, or bleeding in persons of weak, relaxed habits, or on any other pulse than a hard one, produces an irritable state of excitement that favors a typhoid tendency. A teaspoonful of spts. nitri dul. should be given in water, in adult cases, every two hours, during the exacerbation, when the stomach will bear it. During the remission, if there should be any, two teaspoonsful of spts. mindereri may be given every two hours until the exacerbation, when the nitre should be resumed. The surface should be regularly sponged off every few minutes with warm vinegar and water. The head should be frequently wet with cold water, when there is considerable pain with heat in it; when the pain is unattended with great heat, spts. camphor should be used instead of water. Also when there is on the surface generally an acrid or pungent heat, cold water may be used as a wash instead of the warm vinegar and water, if it does not produce a sensation of chilliness. For a drink, soda water may be allowed and cream of tartar water where the bowels are not too active.

To fulfil the second grand indication, we depend on calomel. A small dose of it, say 8 grs., taken at bed time and worked off next day with castor oil, and followed up two or three times if necessary, will probably in most cases break up an attack. When the attack is sudden or the fever completely formed before the physician is called, the doses should be larger, say 15 grs., and followed if necessary by castor oil or rhubarb. Should the operations be watery, stop them immediately by laudanum or opium in substance, and repeat the calomel. And where the operations are consistent, the bowels should be permitted to rest for six hours before the repetition. This course should be unremittingly followed till the secretions in general, and the alvine in particular, become healthy, or the fever is broken. Where there is a bilious purging from the commencement or where it occurs at any time during the course of the fever, I am in the habit of giving, in the place of calomel alone, the following powder: cal. 5 grs., opii. ½ gr., ipecac. ½ gr. or 1 gr., when the stomach will bear it, every three hours until four are taken, following them in four hours after the last with castor oil if necessary, and restraining the operations with laudanum if they should be too large or watery. This course should be repeated every 24 hours till the desired effect is produced.

When this course does not effect a cure and the fever threatens to assume the typhoid type, to fulfil the third indication, a sinapism should be applied to the spine once every 24 hours, to remain long enough each time to make the skin very red. This will stimulate the ganglionic nerves supplying the liver, where they are much oppressed by disease. This application is necessary where the discharges from the bowels are of a dark muddy color and watery. Without it, the internal remedies fail of having their desired effect. Should the fever be of the typhoid type when the physician is called, or should it appear to be assuming that type under his hands, if the tongue is moist enough to allow it, a powder should be given, composed of 3 grs. quinine and 4 grs. calomel, every four hours, until four are taken. They should be followed by oil about five hours after the last powder. This course should be repeated every 36 hours until the fever is broken or the gums are affected by the calomel, when it should be omitted, while the quinine may, if necessary, be continued. I have feared to give the quinine where the tongue is of a dry red colour or black, nor do I recollect seeing it recommended in such cases by any author. In its stead I give carbo ligni, finely pulverized, in 20 gr. doses every four hours, as long as it does not produce unpleasant sensations at the stomach. And every 24 hours, give a course of the camphorated powders, used in the treatment of the congestive variety, at intervals of three hours, following each course by a cathartic if necessary, until the tongue becomes moist and the secretions free. In this stage of the fever stimulants frequently become necessary; and Port or good Madeira wine seems to answer best. They should be used in those cases only where the vital powers of the system rapidly sink. Wine given at short intervals, assisted by stimulating frictions of the surface, will keep up an action long enough to permit the other medicines to affect the secretions in cases which would otherwise prove fatal.

At any stage after the inflammatory diathesis is subdued, when there is a dry tongue of a brown or bright red color, attended with bilious purging, blisters should be applied to the bowels, and to the back of the head and to the ankles when the head is much affected.

Whenever the symptoms of a slight chill introduce such paroxysm, should the tongue be moist, the chill may be broken up by the use of calomel and quinine at intervals of three hours before it is expected. Quinine may be used separately if the purging forbid the use of calomel.

After the secretions become free, and a remission or intermission is fairly established, tonics should be freely used to complete the cure.

And, in conclusion, as tartar emetic might be mentioned as a remedy in this form of fever, I will state the result of my experience respecting it in few words. In the commencement of this form, when bilious matter is frequently vomited, and the presence of bile in the stomach causes a constant sickness, a gentle emetic of tartar is very serviceable, perhaps indispensable. But, given in every case indiscriminately, it produces an irritable state of excitement, which increases a typhoid tendency. Given in either of the preceding varieties, the effects are almost invariably fatal.

November, 1841.

